# Exosomal Long Noncoding RNAs in NSCLC: Dysfunctions and Clinical Potential

**DOI:** 10.7150/jca.84506

**Published:** 2023-06-12

**Authors:** Hongze Lin, Jiaying Li, Maoye Wang, Xu Zhang, Taofeng Zhu

**Affiliations:** 1 Department of Pulmonary and Critical Care Medicine, Yixing Hospital affiliated to Jiangsu University, Yixing 214200, China.; 2 School of Medicine, Jiangsu University, Zhenjiang 212013, China.; 3 Jiangsu Key Laboratory of Medical Science and Laboratory Medicine, Department of Laboratory Medicine, School of Medicine, Jiangsu University, Zhenjiang 212013, China.

**Keywords:** exosome, lncRNA, NSCLC, biomarker, target

## Abstract

Exosomes are a typical subset of extracellular vesicles (EVs) that can be transmitted from parent cells to recipient cells via human bodily fluids. Exosomes perform a vital role in mediating intercellular communication by shuttling bioactive cargos, such as nucleic acids, proteins and lipids. Long noncoding RNAs (lncRNAs) are transcripts longer than 200 nucleotides without protein translation ability and can be selectively packaged into exosomes. Accumulating evidence indicates that exosomal lncRNAs have a critical role in tumor initiation and progression through regulating tumor proliferation, apoptosis, invasion, metastasis, angiogenesis, treatment resistance and tumor microenvironment. Increasing studies suggest that exosomal lncRNAs have great potential to be served as novel targets and non-invasive biomarkers for diagnosis and prognosis in non-small cell lung cancer (NSCLC). In this review, we provide an overview of current research on the disordered functions of exosomal lncRNAs in NSCLC and summarize their potential clinical applications as diagnostic and prognostic biomarkers and therapeutic targets for NSCLC.

## Introduction

Lung cancer is one of the most common malignancies and the leading cause of cancer related death around the world with more than 2 million new patients and 1.76 million deaths annually 1. Non-small cell lung cancer (NSCLC) is the predominant histological subtype of lung cancer, accounting for 85% of lung cancer patients 2, 3. NSCLC can be further divided into three main subtypes: adenocarcinoma, squamous cell carcinoma, large cell carcinoma 4. Due to lack of obvious early symptoms and reliable biomarkers for early diagnosis, patients with lung cancer are often diagnosed at advanced stage associated with dismal prognosis. Although significant advances in diagnosis and treatment for lung cancer have been obtained, the overall 5-year survival rate is as low as 17.9% and the 5-year survival rate for patients with distant metastasis is only 4% 5, 6. Its lethality highlights a need to identify novel insights into the mechanisms, reliable biomarkers and targeted therapy candidates of NSCLC.

Extracellular vesicles (EVs) are a kind of small membrane vesicles with phospholipid bilayer structure 7. EVs can be classified into two main types: exosomes, ectosomes or micro-vesicles on the basis of their biological occurrence and size 8. Unlike ectosomes, exosomes are biological nanoparticles with a size of 40-150nm in diameter 9, which are generated from multivesicular bodies (MVBs) formed from inward budding of endosomes and are released via exocytosis pathway after MVBs fuses to cell membrane 10-12. Exosomes are widely present in a variety of body fluids, such as blood, urine, cerebrospinal fluid, pleural fluid and saliva 13. First detected in sheep reticulocytes in 1983, exosomes were initially thought to be involved with cellular waste cleaning 14. With further research on exosomes going deep, however, exosomes have been demonstrated to play a crucial role in intercellular communication by transmitting cargoes, such as nucleic acids and proteins 15. Importantly, numerous studies reveal that exosomes contained with specific bioactive components participate in regulating tumor progression from diverse aspects, including tumor cell proliferation, apoptosis, invasion, metastasis, angiogenesis 8, 16-19. Cancer-related functions and widespread distribution in human body indicate the potential of exosomes as therapeutic targets and non-invasive biomarkers for diagnosis and prognosis in cancer management. Growing evidence confirms that long noncoding RNA (lncRNA) can be packaged into exosomes and transferred from parent cells to adjacent cells or distant tissues through human circulatory system, thereby modulating biological activities in recipient cells 20-22. An increasing number of exosomes containing lncRNAs are ubiquitous in both bodily fluids and tumor tissues, and are found to be abnormally expressed in a variety of cancers, including NSCLC 23-25. Recent studies have indicated that exosome-derived lncRNAs play an incredible role in tumorigenesis and progression of multiple cancers 26, 27, providing the possibility that exosomal lncRNAs function to be targets for cancer treatment and novel biomarkers for cancer diagnosis and prognosis 28, 29. In this review, we firstly discuss basic features of exosomes and emerging biological functions of lncRNAs. Then, in particular, we focus on the disordered regulatory roles of exosomal lncRNAs and their clinical applications in NSCLC. The existing challenges that hinder the clinical applications of exosomal lncRNAs and future development directions are finally pointed out.

## Biology of exosomes

### Biogenesis and release of exosomes

**Figure 1:**
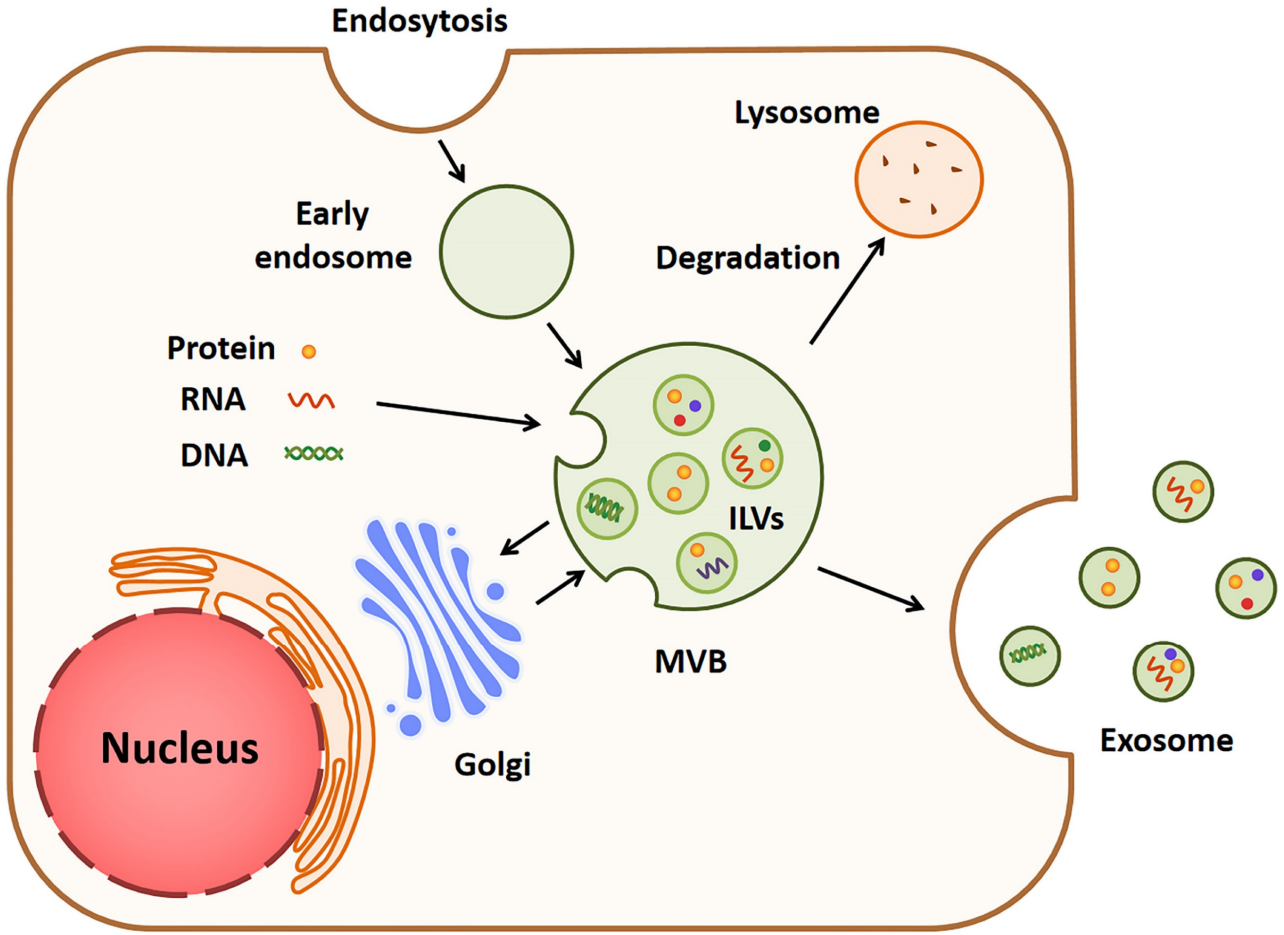
** The biogenesis and release of exosomes.** Cell membrane invaginates to form a vesicle called early endosome, and its membrane then bends inward to form a multi-vesicular body (MVB). MVB sprouts inward to form intracavitary vesicles (ILVs) rich in proteins, RNAs and DNAs. When the MVB is fused to cell surface, ILVs are released into exosomes.

As one of the main types of EVs, exosomes can be released by a majority of cells and play critical roles in cell-to-cell crosstalk under diverse pathophysiological conditions 30. The formation of exosomes is a complex process involving several major steps, which is shown in **Fig. 1**. For the first step, the cell membrane sinks inward and forms vesicles carrying substances from the cell surface and extracellular space. The vesicles then fuse with a cell structure named early endosome and move along special protein tracks 31, 32. During the movement, the membrane of early endosome curves inward, which leads to the formation of smaller vesicles known as multi-vesicular bodies (MVBs) which bud inward to form intraluminal vesicles (ILVs) 33. Multiple components, such as lipids, proteins, microRNAs, lncRNAs and ctDNAs, are enriched in ILVs, while the underlying mechanisms of component sorting have not been fully determined yet. Both endosomal sorting complex required for transport (ESCRT)-dependent and independent manners were reported to interpret this mechanism 34-36. Finally, ILVs are released to become exosomes when the ILVs-loaded MVB fuses with the cell surface 37, 38.

### Functions of exosomes in cancer

Exosomes are a fascinating new research field attracting the attention of many researchers. It was originally thought that exosomes were used to remove unwanted waste produced by cells 31. In recent years, however, it has been clear that exosomes have remarkable roles in modulating pathophysiological activities 39. Ongoing dialogues between cells are present via exosomes to regulate various cancer-related biological activities, including tumorigenesis, progression, metastasis, angiogenesis and drug resistance 40. Exosomes function by transferring multiple bioactive components, including proteins, RNA molecules, DNA, which leads to changes in cellular function of recipient cell 41-43. For example, exosomal miR-21 generated by oral squamous cell carcinoma can be transmitted to normoxic cells to promote tumor metastasis under hypoxic microenvironment 44. Serum-derived exosomal miR-96 enhances the progression of lung cancer by targeting protein LMO7 45. On the other hand, the contents and quantities of exosomes are identified to vary in different diseases, including various cancers. PLA2G10 mRNA and protein levels in serum exosome are significantly increased in NSCLC, which are linked with invasive clinical feature and poor prognosis of NSCLC patients 46. In addition to the abnormal expression of exosome-derived components related to caner, exosomes can cross biological barriers and widely exist in body fluids, suggesting that exosomes may be served as a source of non-invasive indicators of cancers. Exosomal RP5-977B1 has been demonstrated as a novel promising biomarker for diagnosis and prognosis of NSCLC 47. Furthermore, exosomes can be harnessed to deliver medicine with good stability 48. Therapeutic agents mediated by exosomes, like nucleic acids and proteins, can be protected from degradation since exosomes are wrapped by lipid bilayers 49. Combined with special ligands as aptamers, exosomes can target specific molecules or cell types, which can be utilized in precise treatment of cancer 50, 51. A recent study reports that Aptamer-Functionalized Exos (Apt-Exos) are created as an ideal platform for antitumor drug delivery through the combination of exosomes and aptamers that can specifically recognize cell types 52. Exosome-mediated drugs have been under investigation and entered preclinical study stage already 53. In summary, exosomes have great potential in the diagnosis, prognosis and treatment of cancer management.

## Biological functions of lncRNAs

**Figure 2:**
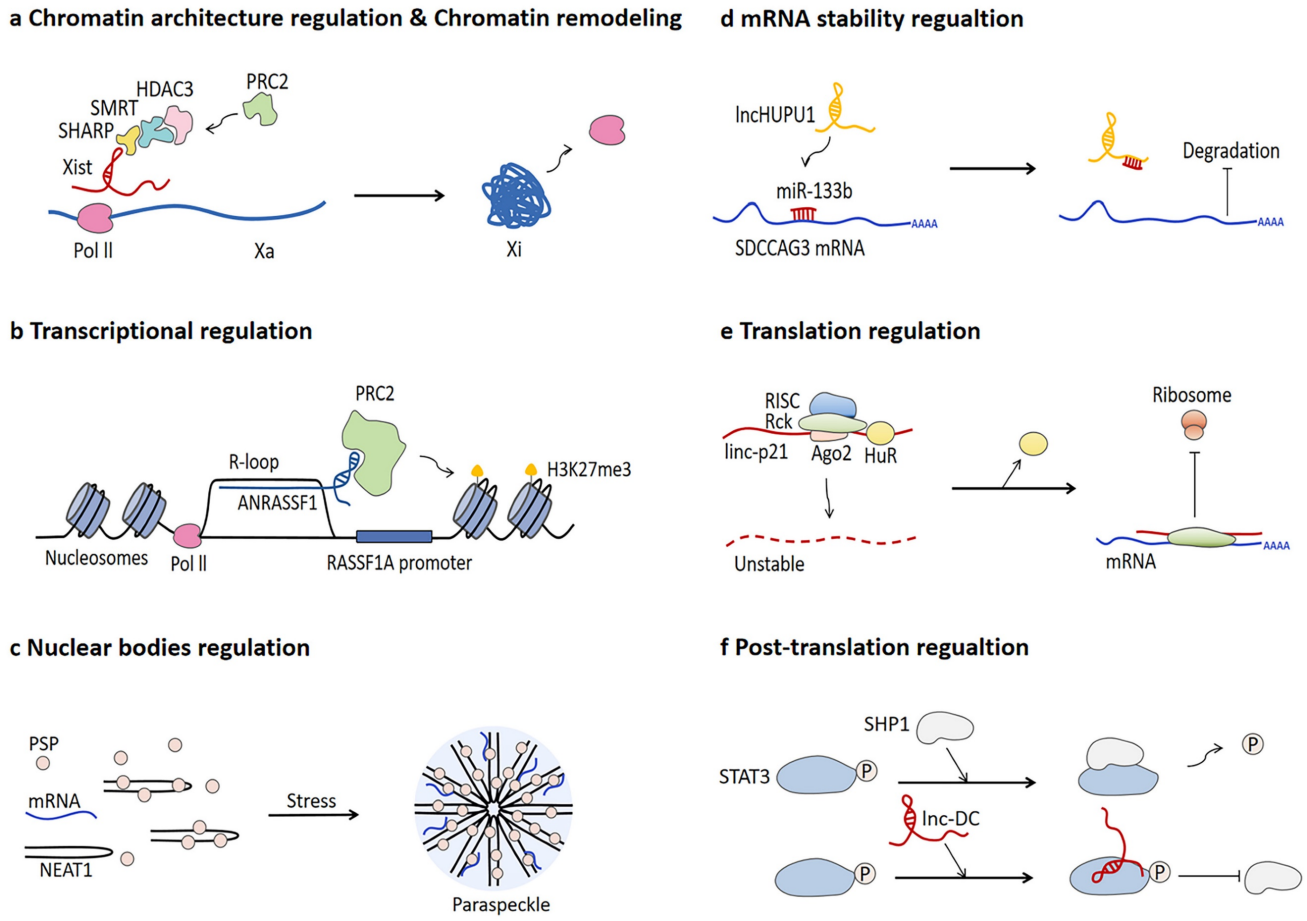
** Biological functions of lncRNAs. a** lncRNA Xist regulates chromatin architecture and chromatin remodeling. **b** lncRNA ANRASSF1 participates in transcriptional regulation by forming R-loop.** c** NEAT1 modulates nuclear bodies by assembly as a scaffold of paraspeckle, especially under stress conditions. **d** lncHUPU1 functions as a competitive endogenous RNA (ceRNA) to regulate mRNA stability. **e** linc-p21 regulates translation process by hindering ribosomes from translating mRNA when HuR is absent. **f** lnc-DC has roles in post-translation regulation through preventing SHP1-mediated STAT3 dephosphorylation.

lncRNA research is a rapidly expanding field of genomics. lncRNAs are a large and diverse class of RNA transcripts longer than 200 nucleotides in length and hardly translated into proteins 54-57. lncRNAs were originally considered as wastes of genome transcription and just by-product of Pol Ⅱ transcription 58, whereas growing evidence confirms that lncRNAs can exert regulatory functions in multiple biological processes, as shown in **Fig. 2**.

The most well-known mechanism of lncRNA-mediated chromatin architecture regulation and chromatin remodeling is related to dosage compensation. lncRNA Xist can recruit various proteins in an orderly manner during the early stage of embryonic development, triggering gene repression of the entire X chromosome, which ultimately leads to chromosome condensation into an inactive Barr body 59-62. Different to lncRNA Xist, CoT-1 RNA is determined to be highly correlated with euchromatin and promote chromatin opening 63. In addition, p53-regulated lncPRESS1 interacts with SIRT6 protein to enrich histone H3K9ac and H3K56ac on the promoters of specific genes related to pluripotency and differentiation of embryonic stem cells and inhibit chromatin remodeling 64. Forming a R-loop characterized by a hybrid structure composed of RNA and double-strand DNA (dsDNA) is one of the mechanisms of lncRNA-mediated transcriptional regulation 65, 66. The R-loop formed by lncRNA ANRASSF1 recruits PRC2 protein to the promoter of RASSF1A gene and subsequently triggers histone H3K27 trimethylation, thereby repressing RASSF1A expression 67. lncRNA also participate in modulating the assembly process and function of nuclear bodies 68. For instance, NEAT1 can function as scaffold of para-speckle to mediate gene expression at the post-transcriptional level 69-72. The number and morphology of para-speckles are positively correlated with the expression of NEAT1, especially under the stress condition of increased NEAT1 expression 73.

lncRNA is capable of regulating the stability of mRNA through diverse mechanisms to affect mRNA expression level. For example, lncHUPC1 functions as competitive endogenous RNAs (ceRNAs) to absorb miR-133b, thereby regulating SDCCAG3 expression in prostate cancer 74. Additionally, lncRNA Cyrano triggers the destruction of miR-7 by binding to miR-7 and then promoting the cleavage and cleavage of the 3' end, leading to the accumulation of circular RNA Cdrlas, a miR-7 target, in the brain 75. The BACEA-antisense transcript (BACEA-AS) promotes the stability of BACE1 mRNA and upregulates BACE1 protein level via base pairing with BACE1 mRNA 76. Moreover, the binding of NORAD to cytoplasmic proteins PUMILIO1 and PUMILIO2 (PUM1/2) limits their effective degradation of target mRNAs 77, 78.

lncRNA is also involved with translation regulation and post-translational regulation 79. When RNA-binding protein HuR is present, linc-p21 tends to recruit RNA-induced silencing complex (RISC) and becomes instable consequently. However, in the absence of HuR, linc-p21 acts as an inhibitor to regulate the translation of target mRNA by reducing polymer size and complementing the base pairs of target mRNAs to cause ribosomes shedding 80. It is also confirmed that lncRNA correlates with the regulation of protein translational modifications (PTMs). lnc-DC has regulatory effect on PTMs regulation by directly binding to STAT3, which inhibits the combination between STAT3 and SHP1 responsible for dephosphorylating STAT3, thereby promoting the phosphorylation of STAT3 81.

## Dysfunctions of exosomal lncRNAs in NSCLC

**Figure 3:**
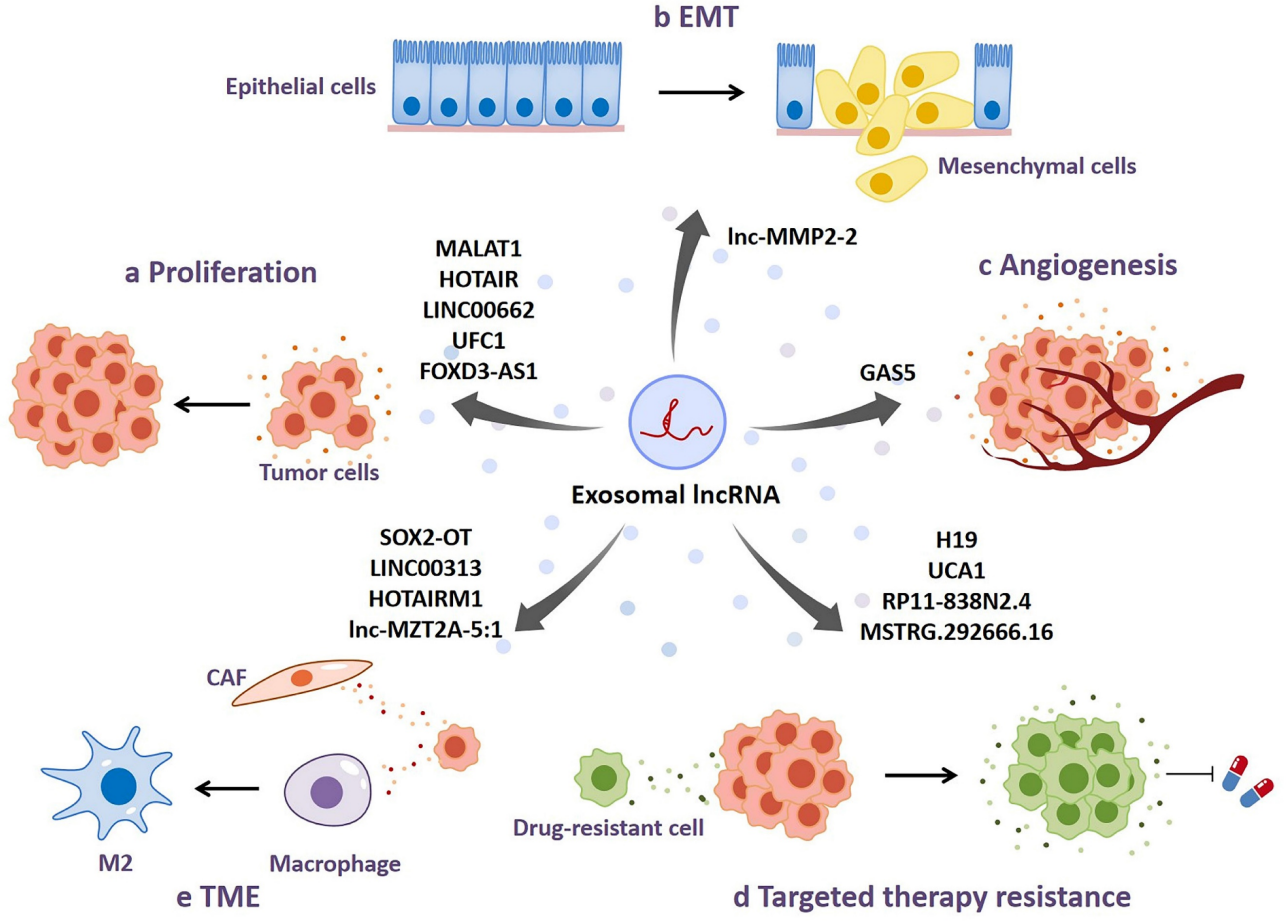
** Dysfunctions of exosomal lncRNAs in NSCLC. a** Exosomal lncRNAs promote tumor proliferation in NSCLC. **b** Exosomal lncRNAs enhance epithelial-mesenchymal transition (EMT) in NSCLC.** c** Exosomal lncRNAs participate in regulating NSCLC angiogenesis.** d** Exosomal lncRNAs regulate targeted therapy resistance in NSCLC. **e** Tumor cell-derived exosomal lncRNAs modulate the biological functions of other cells in tumor microenvironment (TME).

lncRNA can modulate biological activities of recipient cells by being transferred in the form of exosomes. Accumulating evidence indicates that dysfunctions of exosomal lncRNAs occur in a variety of malignancies, which is involved with tumor growth, progression, angiogenesis, and therapy resistance. It is of importance to comprehend the underlying mechanisms how exosomal lncRNAs function to regulate tumor development. In this section, we specially discuss about the dysfunctions of exosome-derived lncRNAs in NSCLC and potential molecular mechanisms, as shown in **Fig. 3** and **Table 1**.

### Proliferation and apoptosis

The promoting function of lncRNA-contained exosomes on tumor proliferation has been widely reported. For instance, exosomes derived from serum samples of NSCLC patients are enriched in metastasis-associated lung adenocarcinoma transcript 1 (MALAT-1) 23. Silencing of MALAT-1 restrains tumor proliferation, invasion and migration, and induces cell apoptosis, leading to repressed tumorigenic process of NSCLC. MALAT-1 may act as a sponge of miR-515-5p to increase EEF2 expression, thereby facilitating tumor growth in NSCLC 82. HOX transcript antisense RNA (HOTAIR) is the first reported lncRNA associated with malignancies. Chen et al. suggest that serum-derived exosomes isolated from NSCLC patients contain increased levels of HOTAIR, which could enhance tumor proliferation and migration and inhibit the apoptosis of NSCLC 83. Zhang et al. further determine that exosomal HOTAIR may be miR-203 sponge to facilitate tumor growth and progression of NSCLC 84. LINC00662-contained exosomes are abundant in plasma samples of NSCLC patients. They can upregulate the expression of E2F transcription factor 1 (E2F1) by interacting with miR-320d, promoting cell proliferation, invasion and migration and hinder apoptosis in NSCLC, which contributes to tumor progression in NSCLC 85. Exosomal lncRNAs can not only act as ceRNAs to modulate NSCLC growth and progression, but bind to tumor-associated proteins to impact signaling pathways critical for NSCLC development. Zang et al. indicate that serum-derived exosomes isolated from NSCLC patients contain the markedly high levels of UFC1, which is one of several specific lncRNAs responsible for recruiting EZH2 histone methyltransferase to silence gene expression in an epigenetically modified manner, resulting in the repressed expression of PTEN and the activated Akt signaling pathway, thereby enhancing NSCLC cell proliferation, cell cycle arrest, invasion and migration, and inhibiting apoptosis 86. Moreover, NSCLC cells can release exosomes containing considerable lncRNA FOXD3-AS1, which is able to stimulate the PI3K/AKT pathway of recipient cells through binding to ELAV-like RNA-binding protein 1 (ELAVL1), resulting in the promoted NSCLC cell proliferation and invasion and the repressed 5-fluorouracil-induced apoptosis 87. Altogether, exosome-derived lncRNAs function as facilitators of NSCLC growth and progression by acting as ceRNAs sponging miRNAs or interacting with key proteins in specific signaling pathways important for mediating cell growth.

### Invasion and metastasis

Invasion and metastasis are critical phases of cancer progression, contributing to cancer mortality in diverse mechanisms 88. Plasma exosomes of metastatic patients contain upregulated lncRNA stem cell inhibitory RNA transcript (SCIRT), which is associated with the advanced stage of NSCLC 89. Instead of directly promoting cancer progression, intriguingly, lncRNA SCIRT selectively sorted miR-665 into cancer-derived exosomes in a hnRNAPA1-dependent manner, and subsequently exosomes enriched with miR-665 play a direct role in enhancing NSCLC invasion and migration through targeting Notch downstream transcription factor HEYL. Epithelial-mesenchymal transition (EMT) has been demonstrated as an important part of inducing metastasis and promoting tumor progression, which is characterized by reduced adhesion between epithelial cells, enabling tumor cells to prone to metastasis 90. Wu et al. investigate that lnc-MMP2-2 is enriched in exosomes extracted from NSCLC cells pretreated with transforming growth factor (TGF)-β, a potent inducer of EMT 91. In accordance with bioinformatics analysis that indicated lnc-MMP2-2 as a positive regulator of target gene expression at transcriptional level, exosomal lnc-MMP2-2 may facilitate the progression of NSCLC by activating the expression of its upstream gene MMP2 which is positively correlated with invasion and vascular permeability of lung cancer when entering recipient cells 91-95. The results of overexpressing and silencing lnc-MMP2-2 in human brain microvascular endothelial cells (HBMECs) further identifies that lnc-MMP2-2 can impair tight junctions between HBMECs and induce EMT, resulting in the increased permeability of blood-brain barrier (BBB) and thus, enabling cancer cells in circulating system to penetrate into the brain 96. Mechanistically, lnc-MMP2-2 may serve as a ceRNA to sponge miR-1207-5p in recipient cells after exosome uptake, thereby directly increasing the expression of EPB41L5 responsible for promoting NSCLC metastasis. In addition to brain metastasis, bone metastasis is one of the common cases of NSCLC distant metastasis. Peripheral blood-derived exosomes isolated from NSCLC patients with bone metastasis contain high levels of SOX2 overlapping transcript (SOX2-OT), which is closely related to poor overall survival and can promote bone metastasis in NSCLC by increasing the expression of RAC1 via targeting miR-194-5p 97. Although the absence of RAC1 has been shown to repress osteoblast differentiation, it is unclear how RAC1 plays an important role in the TGF-β/pTHrP/RANKL signaling pathway, the best-known mechanism of osteolytic metastasis 98, 99. Collectively, these findings suggest that exosmal lncRNAs-mediated intercellular cross-talk mediated may play an important role in regulating the invasion and metastasis of NSCLC.

### Angiogenesis

Angiogenesis is an important condition for tumorigenesis, growth and progression. Increasing evidence shows that exosomes from tumor cells participate in regulating tumor angiogenesis through diverse mechanisms 100-103. Cheng et al. determine that human umbilical vein endothelial cells (HUVECs) treated with exosomes secreted form NSCLC cells exhibit enhanced cell proliferation and tube formation and inhibited apoptosis 104. They further prove that exosomes secreted by NSCLC cells contain reduced levels of lncRNA growth arrest specific 5 (GAS5), which upregulates the expression of key lung cancer angiogenesis protein PTEN by binding to miRNA-29-3p and inhibits the PI3K/AKT signaling pathway of HUVECs, thus exerting its anti-angiogenesis effect on HUVECs 104-106. Exosomes containing high expression levels of GAS5 can inhibit cell proliferation and tube formation and promote the apoptosis of HUVECs, contributing to repressed angiogenesis and tumor metastasis 104. Therefore, exosomal lncRNAs may be considered as important mediators for NSCLC angiogenesis.

### Targeted therapy resistance

With the in-depth research of tumor-related mechanisms and the development of cancer treatment, tumor targeted therapy has become a hot spot, the application of which in NSCLC patients has achieved notable efficacy. However, acquired resistance to tumor targeted therapy inevitably occurs after initial effective response, leading to inferior therapeutic effect and poor prognosis 107, 108. Thus, it is of necessity to unveil the underlying mechanisms and to discover potential biological targets that have significant roles in drug resistance of tumor targeted therapy. Increasing studies have shown that exosomes are involved in tumor targeted therapy resistance in diverse manners. Notably, exosomes can transfer functional molecules, including lncRNAs, from drug resistant cells to sensitive cells, stromal cells to tumor cells, resulting in drug resistance transmission 109, 110.

lncRNA H19 is a widely accepted oncogene in multiple cancers. Its role in NSCLC targeted therapy resistance has been evaluated, which indicates that H19 can act as a facilitator of resistance to gefitinib, a representative first-generation tyrosine kinase inhibitor, by hnRNPA2B1-mediated incorporation in exosomes 111. NSCLC cells treated with exosomes containing high H19 levels exhibit significantly increased resistance to gefitinib, while the results of H19 silencing are reversed. Moreover, Chen et al. reveal that gefitinib-resistant NSCLC cells release exosomes containing the elevated expression of lncRNA urothelial carcinoma-associated 1 (UCA1), which can promote gefitinib resistance by modulating FOSL2 expression via binding to miR-143 112. Dissertation of cell phenotypes and animal experiments suggests that UCA1 may play a regulatory role in facilitating gefitinib resistance, and UCA1 knockdown contributes to inhibited cell proliferation and enhanced cell apoptosis induced by gefitinib. In addition to modulating gefitinib resistance, lncRNAs can also have effect on resistance to other targeted drugs. For instance, exosomal lncRNA RP11-838N2.4 is highly expressed in serum samples of erlotinib-resistant patients 113. Erlotinib-sensitive cells treated with exosomes containing lncRNA RP11-838N2.4 are transformed into erlotinib-resistant cells, whereas lncRNA RP11-838N2.4 downregulation reverses the effect, suggesting that exosomes may disseminate erlotinib resistance by delivering lncRNA RP11-838N2.4 from erlotinib-resistant cells to erlotinib-sensitive cells in NSCLC. Furthermore, the association between lncRNA MSTRG.292666.16 and osimertinib resistance has also been under investigation, indicating that lncRNA MSTRG.292666.16-contained exosomes induce acquired resistance to osimertinib in NSCLC. lncRNA MSTRG.292666.16 is enriched in exosomes derived from osimertinib-resistant plasma compared with those secreted by osimertinib-sensitive plasma 114. Its knockdown impaires the resistance of H1975R cells to osimertinib. Overall, exosome-derived lncRNAs are involved in acquired targeted therapy resistance during the management of NSCLC patients, which offers a new idea for considering exosomal lncRNAs as therapeutic targets for NSCLC patients with targeted therapy resistance.

### Cross-talk in tumor microenvironment

Tumor microenvironment (TME), a complex integrated system which is composed of tumor cells, inflammatory cells, immune cells, stromal cells, other cellular components and non-cellular components 115. In the process of tumor development, there are a variety of dialogues between tumor cells and other cells in TME 116-118. Increasing studies have determined that tumor cells regulate the biological functions of other cells in TME through secreting exosomes containing lncRNAs.

Tumor-associated macrophages (TAMs) are the main tumor-infiltrating immune cells, which are usually induced by tumor cells to promote tumor immune escape, tumor growth, metastasis, drug resistance and angiogenesis 119. NSCLC cells can promote M2 macrophage polarization through transferring exosomes containing increased LINC00313 120. After being taken by macrophages, exosomal LINC00313 can competitively bind to miR-135a-3p to increase STAT6 expression, promoting tumor progression. In addition, tumor cell-derived exosomal SOX2-OT can also be transferred into macrophages and facilitate M2 macrophage polarization and repress M1 polarization by sponging miR-627-3p to upregulate Smads expression, ultimately enhancing EGFR-TKIs resistance in NSCLC 121. On the other hand, the exosomal lncRNA-related crosstalk between tumor cells and cancer associated fibroblasts (CAFs) in TME has been explored. Exosomal HOTAIRM1 secreted by NSCLC cells can be transferred into CAFs and interact with miR-328-5p to increase the expression of SPON2 in CAFs cells, contributing to the development of NSCLC 122. Interestingly, exosomal lnc-MZT2A-5:1 derived from osimertinib-resistant NSCLC cells can activate fibroblasts and promote the migration and inflammation of fibroblasts 123. Collectively, these tumor cells-derived exosomal lncRNAs may play an important role in TME and become potential therapeutic targets for NSCLC treatment.

## Clinical potential of exosomal lncRNAs in NSCLC

Although tissue biopsy is the gold standard for NSCLC diagnosis in clinical practice, the method has several limitations, such as tissue volume, repeatability and inability to characterize tumor heterogeneity, resulting in a lack of an accurate and complete disease status diagram 124. In addition, tissue biopsy may increase the possibility of tumor metastasis through invasive operation, leading to tumor progression and shorter survival 125. However, in comparison with tissue biopsy, liquid biopsy offers an alternative for early diagnosis and real-time monitoring of tumor patients in a non-invasive and more comprehensive manner 126, 127. Exosomes abundantly present in almost bodily fluids and contain a variety of bioactive molecules that can reflect dynamic tumor status due to their origin. The value of exosomal miRNAs on diagnosis and prognosis of malignancies has been demonstrated extensively. Nevertheless, emerging evidence supports that lncRNAs perform better disease-specific expression compared with miRNAs 128, 129. Increasing studies have proven the role for exosomal lncRNAs as circulating biomarkers for diagnosis and prognosis and therapeutic targets in NSCLC 130. In this section, we summarize the clinical potential of exosomal lncRNAs in NSCLC, as shown in **Table 2**.

### Diagnostic biomarkers

Due to remained poor prognosis of NSCLC patients, early diagnose of NSCLC before major clinical events is of great importance, which can effectively reduce the related mortality. Several exosomal lncRNAs have been demonstrated as novel diagnostic biomarkers in NSCLC. Compared with healthy controls, serum exosomal lncRNA GAS5 is significantly downregulated in NSCLC patients, and its low expression is associated with advanced TNM stage and larger tumor size of NSCLC patients 131. Receiver operating characteristic (ROC) curve analysis reveals that the diagnostic performance of exosomal lncRNA GAS5 (area under the curve (AUC) of 0.857) is superior to that of carcinoembryonic antigen (CEA) (AUC of 0.758), and the combination of both biomarkers increases the AUC of 0.929. Notably, exosomal lncRNA GAS5 holds the potential to be a reliable indicator for early detection of NSCLC, because it can distinguish stage I NSCLC patients from healthy individuals with an AUC of 0.822. Exosome-derived DLX6-AS1 also presents as a novel diagnostic biomarker of NSCLC. DLX6-AS1 functions as an oncogene in multiple solid tumors, including NSCLC. Consistent with the expression in serum, the expression of DLX6-AS1 in serum exosomes of NSCLC patients is markedly higher than that of healthy subjects 132. In contrast with circulating CYFRA21-1, a serum marker for NSCLC, circulating DLX6-AS1 has higher sensitivity and specificity in NSCLC diagnosis through ROC curve analysis, and the AUC of circulating DLX6-AS1 and CYFRA21-1 are 0.806 and 0.600, respectively. Exosomal LINC00917 is considered as a potential candidate of NSCLC diagnosis. In line with its expression pattern in human colorectal cancer, LINC00917 is significantly enriched in exosomes derived from serum samples of NSCLC patients and much higher in advanced NSCLC patients (Stage Ⅲ/Ⅳ) than in NSCLC patients with early stages (Stage Ⅰ/Ⅱ) 133. Importantly, ROC curve analysis shows that exosomal LINC00917 performs a good AUC value (0.811) in distinguishing NSCLC patients from healthy controls. Compared with certain NSCLC biomarkers, including CEA, CYFRA21-1and CA-125, exosomal LINC00917 has better diagnostic value for patients with advanced NSCLC (Stage III/IV) with an AUC of 0.907, suggesting that exosomal LINC00917 tends to be a specific biomarker for detecting patients with advanced NSCLC. Cox regression model further demonstrates its diagnostic value, revealing exosomal LINC00917 in high level significantly correlates with short overall survival (OS) of NSCLC patients.

Exosomal lncRNA TBILA and AGAP2-AS1 are suggested as potent biomarkers for diagnosing NSCLC 134. Compared with healthy subjects, the levels of exosomal lncRNA TBILA and AGAP2-AS1 in serum from NSCLC patients are relatively high, while the levels of both decrease after surgery. The correlation between two exosomal lncRNAs and clinical characteristics is explored, highlighting that exosomal lncRNA TBILA is involved with tumor size and exosomal lncRNA AGAP2-AS1 is associated with lymph node metastasis and TNM stage. ROC curve analysis results verify the diagnostic efficacy of two exosomal lncRNAs for NSCLC, showing that the AUC values of exosomal lncRNA TBILA and AGAP2-AS1 are 0.775 and 0.734, respectively. Although combining these two exosomal lncRNAs is not able to improve diagnostic efficiency, the integration of them and Cyfra21-1 can achieve optimal NSCLC diagnostic accuracy. It is noteworthy that exosomal lncRNA TBILA (sensitivity of 63.2%) is more sensitive in discriminating early stage of NSCLC than exosomal lncRNA AGAP2-AS1 (sensitivity of 42.1%) and Cyfra21-1 (sensitivity of 36.3%). Moreover, a new multiplex detection based on multicolor fluorescence digital PCR EV-lncRNA discovers the increased expression of exosome-derived SLC9A3-AS1 and PCAT6 in peripheral blood of lung cancer patients 135. Given their elevated levels in peripheral blood, the discrimination capability of exosomal SLC9A3-AS1 and PCAT6 is further assessed by ROC curve analysis, revealing that the AUC of them is 0.760 and 0.705 respectively and the combination of them provides a better diagnostic efficiency with an AUC of 0.811, implicating that they are likely to be used as promising biomarkers for lung cancer diagnosis. Exosomal HOTAIR has been demonstrated as a promoter of NSCLC proliferation and migration 83, 84. Its diagnostic performance for NSCLC is evaluated with a high sensitivity of 88.9%, a specificity of 78.3% and an AUC of 0.821, supporting exosomal HOTAIR as novel non-invasive diagnostic marker for NSCLC. There is a significant clinicopathological correlation between the upregulated exosomal HOTAIR and TNM stage as well as lymph node metastasis 83.

Lung squamous cell carcinoma (LSCC) is one of the pathological types of NSCLC. Exosomal SOX2-OT appears to be a reliable biomarker for LSCC diagnosis. Teng et al. indicate that the expression level of exosomal SOX2-OT is significantly upregulated in plasma of LSCC patients and descended after surgical resection of LSCC patients 136. According to ROC curve analysis, the AUC of exosomal SOX2-OT (AUC of 0.815) is higher than that of CEA and squamous carcinoma antigen (SCC), a classic marker for LSCC, and the combined application of exosomal SOX2-OT and serum SCC can obtain a better diagnostic efficacy for LSCC with an AUC of 0.864. Furthermore, exosomal SOX2-OT is positively correlated with clinicopathological parameters, including tumor size, TMN stage and lymph node metastasis.

Overall, all these findings suggest that exosomal lncRNAs are a class of promising biomarkers for NSCLC diagnosis. More research is needed to confirm the diagnostic value of these molecules before real clinical application.

### Prognostic biomarkers

Han et al. estimate the clinical potential of exosomal lncRNA SNHG15 for early diagnosis and prognosis of NSCLC 137. The expression level of serum exosomal lncRNA SNHG15 in NSCLC patients is significantly higher than that in patients with benign lung lesions and healthy controls, but its expression level decreased after tumor resection. The increased level of exosomal lncRNA SNHG15 is positively associated with lymph node metastasis, advanced TNM stage and low differentiation. Univariate analysis shows that NSCLC patients with higher serum exosomal lncRNA SNHG15 levels have poorer prognosis and shorter OS. Multivariate analysis further confirms that exosomal lncRNA SNHG15 is an independent indicator of NSCLC prognosis. According to ROC analysis, the AUC of exosomal lncRNA SNHG15 used alone for differentiating NSCLC from normal controls is 0.856, while the AUC of exosomal lncRNA SNHG15 combined with CEA for early diagnosis of NSCLC increases to 0.915. Collectively, exosomal lncRNA SNHG15 may be considered as a promising candidate for the diagnosis and prognosis of NSCLC. In addition, based on RNA-sequencing analysis of aberrant expression profile of serum exosomal lncRNAs, it is determined that exosomal linc01125 is markedly elevated in NSCLC patients as compared with pneumonia control group 138. The high expression level of exosomal linc01125 is related to advanced T stage and unfavorable OS in NSCLC patients, suggesting that exosomal linc01125 may have the ability to predict the prognosis of NSCLC. ROC analysis is used to evaluate the diagnostic advantage of exosomal linc01125 in discriminating NSCLC from tuberculosis and disease-free controls with the AUC of 0.624 and 0.662, respectively. Overall, exosomal linc01125 may be a potential biomarker for NSCLC diagnosis and prognosis. Min et al. indicate that exosomal lncRNA RP5-977B1 is expected to be a novel non-invasive biomarker for early diagnosis and prognosis of NSCLC 47. Real-time reverse transcription-PCR (qRT-PCR) verifies that its expression level in serum of NSCLC patients is higher than that of healthy controls. NSCLC patients with high levels of exosomal lncRNA RP5-977B1 tend to bear advanced tumor stage, distant metastasis and short overall survival, implicating the capacity of exosomal lncRNA RP5-977B1 to predict the poor prognosis of NSCLC. In accordance with ROC curve analysis, the AUC of exosomal lncRNA RP5-977B1 in discriminating NSCLC from healthy controls and patients with pulmonary tuberculosis is 0.8899 superior to CEA (AUC of 0.7609) and CYFRA21-1 (AUC of 0.6703), and its differential efficiency for early NSCLC is better than both conventional markers. Moreover, plasma exosomal lncRNA HAGLR can be an available indicator for predicting NSCLC-related recurrence and metastasis because of the positive correlation between the expression of exosomal lncRNA HAGLR and lymph node metastasis and TMN stage 139. Intriguingly, the elevated level of exosomal HAGLR is associated with the increased detection rate of circulating tumor cells (CTCs), implying that the combined application of multiple biomarkers may be more helpful in predicting NSCLC prognosis 139.

In conclusion, exosome-derived lncRNAs have great potential in providing new insights into NSCLC diagnosis and prognosis dependent on liquid biopsy. Although the current understanding of exosomal lncRNAs-related liquid biopsy is more in-depth than before, molecular research, large-sample multicenter retrospective and prospective studies are still warranted to verify its clinical value. Of note, the final cut-off value of exosomal lncRNA also needs to be further clarified.

### Therapeutic targets

Although chemotherapy following surgical resection remains a standardized treatment for NSCLC, the overall survival of patients with advanced NSCLC has not been prolonged. Thus, it is necessary to explore novel therapeutic approach. Emerging evidence confirms that exosome-derived lncRNAs play vital roles in mediating targeted treatment of NSCLC, indicating the potential of exosomal lncRNAs as therapeutic targets for NSCLC. lnc-MMP2-2 contained in TGF-β-mediated exosomes promotes the invasion and migration of NSCLC by regulating MMP2 expression, and enhances the brain metastasis of NSCLC by increasing BBB permeability, suggesting that exosomal lnc-MMP2-2 may be a novel therapeutic target for NSCLC treatment 91, 96. Lei et al. reveal that H19 promotes gefitinib resistance in NSCLC through exosome packaging with the assistance of hnRNPA2B1, and propose that exosomal H19 may be a promising target for NSCLC treatment 111. In addition, since exosomal LINC00662 plays an important role in enhancing the development of NSCLC by regulating miR-320d/E2F1 axis, it may serve as a potential therapeutic target for NSCLC patients 85. Exosomal UCA1 can enhance gefitinib resistance in NSCLC by increasing FOSL2 expression via sponging miR-143, which contributes to the limited therapeutic efficacy of epidermal growth factor receptor (EGFR) tyrosine kinase inhibitor (TKI) 112. Meanwhile, it is also found that exosomal SOX2-OT can promote the resistance to EGFR-TKIs in NSCLC cells through binding to miR-627-3p 121. Zheng et al. believe that exosome-derived lncRNA RP11-838N2.4 can be used as a potential target for NSCLC treatment as its participation in inducing erlotinib resistance in NSCLC 113. Osimertinib-resistant NSCLC cells can induce the activation of fibroblasts by transferring exosomes containing lnc-MZT2A-5:1, which is considered to be a novel target for NSCLC treatment 123. In conclusion, targeting exosomal lncRNAs will improve the efficiency of targeted therapy, leading to the prolonged overall survival of NSCLC patients.

## Challenges and future direction

In contrast to free lncRNAs in the circulating system, lncRNAs loaded into exosomes are more stable and freer from the degradation by RNA enzymes. Exosomes wrapped by lipid bilayer membranes which is capable of protecting the loaded drugs from degradation can be considered as natural drug delivery carriers 140. The intrinsic features of exosomes, including unique biocompatibility, tumor targeting capability, high stability and long half-life in human circulating system, enable them to have application value in future tumor therapy 141, 142. Accumulating evidence has identified that exosome-derived lncRNAs play an important role in mediating tumor occurrence, tumor progression, and drug resistance in NSCLC, a portion of which function to be therapeutic targets and available biomarkers for early diagnosis and prognosis of NSCLC with optimal sensitivity and specificity 143. Nevertheless, the following challenges still need to be addressed before the clinical application of exosomal lncRNA.

First, it is difficult to isolate exosomes from human body fluids. So far, there is still no standardized technique for exosome isolation in a high purity and efficient manner, and the core challenge mainly comes from high heterogeneity in size of exosome 144. In this regard, the current exosome separation method is primarily ultracentrifugation which is cost-effective, whereas it also has some defects such as long time-consuming and low extraction efficiency, which makes it difficult to be widely used in clinical practice 145. In addition to ultracentrifugation as the recent main exosome isolation method, a variety of conventional and novel methods, including differential centrifugation, size exclusion chromatography, immunoaffinity capture, precipitation, and microfluidics technique, have their own drawbacks as well 146. Interestingly, the microfluidic device can extract exosomes in an ultra-fast separation and high yield manner, providing a novel alternative for exosome isolation 144. Meanwhile, the storage conditions of exosomes remain an obstacle to exosome-based treatment. The surface and morphological characteristics of exosomes are destroyed under improper storage conditions 147.

Exosomal lncRNAs can be decorated to carry specific sequences or molecules functioning as tumor suppressors, achieving precise treatment of NSCLC with the assistance of nanotechnology, which has great clinical application potential. However, the therapeutic efficiency and safety of exosomal lncRNAs-mediated drug treatment system in NSCLC have not been fully investigated. Once injected into the human body, exosomal lncRNAs may cause cytotoxicity, which involves major safety issues 148. On the other hand, due to the heterogeneous substances contained in exosomes, the imported exosomes may induce immunogenicity in a parental cell-dependent manner 149.

Although a variety of exosome-derived lncRNAs have been proved to play a crucial role in the occurrence and development of NSCLC, there is still a lack of large-scale prospective studies 150. In addition, tumor-specific exosomal lncRNAs have not been determined in NSCLC. The underlying mechanisms of exosomal lncRNAs in the development of NSCLC still needs to be further explored to explain their functions and provide insights for their clinical applications in NSCLC.

## Conclusion

In recent years, remarkable progress in our understanding of exosome-derived lncRNAs has been made, and a clearer landscape of the characteristics and versatile functions of these molecules is emerging. Tumor-related regulatory processes, non-invasiveness, and real-time assessment of tumor status endow exosomal lncRNAs with considerable potential as reliable diagnostic biomarkers and for dynamic monitoring of disease progression and therapeutic intervention. In this review, we primarily summarize the dysfunctions and clinical value of exosomal lncRNAs in NSCLC, and highlight the main challenges that hinder their clinical application. In fact, only a small fraction of exosome-derived lncRNAs have been studies. Therefore, more rigorous research is needed to elucidate how exosomal lncRNAs affect the complicated physiological processes in NSCLC, so as to more comprehensively understand their acting patterns. Addressing relative challenges in the coming years will bring robust diagnosis and prognosis, effective and precise treatment associated with optimal outcomes of NSCLC patients, in an exosomal lncRNAs-dependent manner.

## Figures and Tables

**Table 1:** Dysfunctions of exosomal IncRNAs in NSCLC.

Exosomal lncRNAs	Sample	Origin	Expression	Function	References
MALAT1	Serum, culture medium	NSCLC cells	Increased	Promote proliferation, invasion and migration, and reduce apoptosis	23, 82
HOTAIR	Serum, culture medium	Lung cancer cells	Increased	Promote proliferation, invasion and migration	83, 84
LINC00662	Plasma	NSCLC cells	Increased	Promote proliferation, cell cycle arrest, invasion, and migration, and inhibit apoptosis	85
UFC1	Serum	NSCLC cells	Increased	Promote proliferation, cell cycle arrest, invasion, and migration, and inhibit apoptosis	86
FOXD3-AS1	Culture medium	NSCLC cells	Increased	Promote proliferation and invasion, and inhibit apoptosis caused by 5-FU	87
SCIRT	Malignant pleural effusions (MPEs), plasma, culture medium	NSCLC and SCLC cells	Increased	Promote invasion and migration	89
lnc-MMP2-2	Culture medium	TGF-β-mediated NSCLC cells	Increased	Promote invasion, migration and brain metastasis, and reduce permeability of vascular endothelial cells	91-96
SOX2-OT	Peripheral blood	NSCLC cells	Increased	Promote invasion, migration, bone metastasis and facilitate M2 macrophage polarization	97, 121
GAS5	Serum, culture medium	NSCLC cells	Decreased	Promote tumor angiogenesis	104-106
H19	Culture medium	NSCLC cells	Increased	Promote gefifitinib resistance	111
UCA1	Culture medium	Gefitinib-resistant NSCLC cells	Increased	Promote gefifitinib resistance	112
RP11-838N2.4	Serum, culture medium	Eelotinib-resistant NSCLC cells	Increased	Promote erlotinib resistance	113
MSTRG.292666.16	Plasma, culture medium	Osimertinib-resistant NSCLC cells	Increased	Promote osimertinib resistance	114
LINC00313	Culture medium	NSCLC cells	Increased	Promote M2 macrophage polarization	120
HOTAIRM1	Culture medium	NSCLC cells	Increased	Be associated with CAFs	122
lnc-MZT2A-5:1	Culture medium	Osimertinib-resistant NSCLC cells	Increased	Promote the migration and inflammation of fibroblasts	123

**Table 2:** Clinical potential of exosomal lncRNAs in NSCLC.

Exosomal lncRNAs	Sample	Group	Expression	Role	References
GAS5	Serum	NSCLC (n = 64) vs. healthy controls (n = 40)	Decreased	Diagnostic biomarker	131
DLX6-AS1	Serum	NSCLC (n = 72) vs. healthy controls (n = 64)	Increased	Diagnostic biomarker	132
SOX2-OT	Plasma	LSCC (n = 75) vs. negative controls (n = 79)	Increased	Diagnostic biomarker and therapeutic target	121, 136
LINC00917	Serum	NSCLC (n = 179) vs. healthy controls (n = 104)	Increased	Diagnostic biomarker	133
TBILA and AGAP2-AS1	Serum	NSCLC (n = 150) vs. healthy controls (n = 150)	Increased	Diagnostic biomarker	134
SLC9A3-AS1 and PCAT6	Peripheral blood	Lung cancer (n = 32) vs. healthy controls (n = 30)	Increased	Diagnostic biomarker	135
HOTAIR	Serum	NSCLC (n = 32) vs. healthy controls (n = 20)	Increased	Diagnostic biomarker	83
SNHG15	Serum	NSCLC (n = 118) vs. benign pulmonary lesions (n = 40) vs. healthy controls (n = 80)	Increased	Diagnostic and prognostic biomarker	137
LINC01125	Serum	NSCLC (n = 6) vs. pneumonia controls (n = 5)	Increased	Diagnostic and prognostic biomarker	138
		NSCLC (n = 63) vs. tuberculosis (n = 59)			
		NSCLC (n = 150) vs. disease-free controls (n = 187)			
		NSCLC (n = 62) vs. disease-free controls (n = 95)			
RP5-977B1	Serum	NSCLC (n = 105) vs. healthy controls (n = 51)	Increased	Diagnostic and prognostic biomarker	47
HAGLR	Plasma	NSCLC (n = 40) vs. healthy controls (n = 8)	Decreased	Prognostic	139
lnc-MMP2-2	Culture medium	TGF-β-treated vs. non-TGF-β-treated A549 cells	Increased	Therapeutic target	91, 96
H19	Culture medium	Gefitinib‑resistant cells vs. sensitive parent cells	Increased	Therapeutic target	111
LINC00662	Plasma	/	Increased	Therapeutic target	85
UCA1	Culture medium	Gefitinib resistant vs. Gefitinib-sensitive	Increased	Therapeutic target	112
RP11‑838N2.4	Serum, culture medium	Erlotinib-resistant cells vs. normal NSCLC cells	Increased	Therapeutic target	113
lnc-MZT2A-5:1	Culture medium	Osimertinib-resistant cells vs. normal NSCLC cells	Increased	Therapeutic target	123
